# Single-cell transcriptomic analysis uncovers the origin and intratumoral heterogeneity of parotid pleomorphic adenoma

**DOI:** 10.1038/s41368-023-00243-2

**Published:** 2023-09-07

**Authors:** Xiuyun Xu, Jiaxiang Xie, Rongsong Ling, Shengqi Ouyang, Gan Xiong, Yanwen Lu, Bokai Yun, Ming Zhang, Wenjin Wang, Xiqiang Liu, Demeng Chen, Cheng Wang

**Affiliations:** 1 Hospital of Stomatology, Sun Yat-sen University, Guangzhou, China; 2 Guangdong Provincial Key Laboratory of Stomatology, Sun Yat-sen University, Guangzhou, China; 3 Guanghua School of Stomatology, Sun Yat-sen University, Guangzhou, China; 4 Institute for Advanced Study, Shenzhen University, Shenzhen, China; 5 Department of Oral and Maxillofacial Surgery, Nanfang Hospital, Southern Medical University, Guangzhou, China; 6 Center for Translational Medicine, The First Affiliated Hospital, Sun Yat-sen University, Guangzhou, China

**Keywords:** Cancer, Medical research

## Abstract

Pleomorphic adenoma (PA) is the most common benign tumour in the salivary gland and has high morphological complexity. However, the origin and intratumoral heterogeneity of PA are largely unknown. Here, we constructed a comprehensive atlas of PA at single-cell resolution and showed that PA exhibited five tumour subpopulations, three recapitulating the epithelial states of the normal parotid gland, and two PA-specific epithelial cell (PASE) populations unique to tumours. Then, six subgroups of PASE cells were identified, which varied in epithelium, bone, immune, metabolism, stemness and cell cycle signatures. Moreover, we revealed that CD36^+^ myoepithelial cells were the tumour-initiating cells (TICs) in PA, and were dominated by the PI3K-AKT pathway. Targeting the PI3K-AKT pathway significantly inhibited CD36^+^ myoepithelial cell-derived tumour spheres and the growth of PA organoids. Our results provide new insights into the diversity and origin of PA, offering an important clinical implication for targeting the PI3K-AKT signalling pathway in PA treatment.

## Introduction

Pleomorphic adenoma (PA), as the most common tumour occurring in salivary glands, accounts for two-thirds of all salivary gland lesions, especially in the parotid gland.^1^ It is a benign neoplasm but has a high recurrence rate and the potential for malignant transformation.^2^ The term “pleomorphic adenoma” is defined due to variable cytomorphological and architectural features, which mainly contain three components: epithelial cells, myoepithelial cells and mesenchymal-like cells. Pathologically, PA presents diverse appearances with different ductal epithelial cells and myoepithelial cells growing in a variety of patterns embodied in mucoid-like tissue, myxoid-like tissue, and chondroid-like tissue, indicating that a huge intratumor heterogeneity exists. Consistent with these findings, intratumor molecular heterogeneity has been observed by evaluating the loss of heterozygosity and *PLAG1* gene rearrangements in PAs.^3,4^ Due to diverse tissue architecture and intratumor heterogeneity, the cellular origin in PAs containing morphologically distinct components has been a controversial issue. Interestingly, a previous study showed that both the epithelial and mesenchymal elements were monoclonal using clonal analysis based on random inactivation of one of two x-chromosomes by methylation, suggesting that the original cells for both elements are identical.^5^ Lee et al. further confirmed that both stromal and epithelial cells in PAs arose from the same origin using a human androgen receptor gene (HUMARA) assay.^6^ Notably, recent studies have shown that epithelial cells or myoepithelial cells can transdifferentiate into mesenchymal cells in PAs, suggesting that epithelial-mesenchymal transition (EMT) might be the basic principle of tissue heterogeneity in PAs.^7–10^ These findings support the notion that PA is a monoclonal tumour with a pure epithelial origin despite its diverse intratumor heterogeneity and complex tissue architecture. However, the intratumor heterogeneity of PAs is still largely unknown and there is no convincing experimental evidence to clarify the origin of PAs.

Here, we described a complete atlas of PA and uncovered its cellular complexity and intratumor heterogeneity at single-cell resolution. Our data revealed the transcriptomic profiles of the multicellular ecosystem of PA and showed that PA included acinar, ductal and basal/myoepithelial cells similar to normal parotid glands, along with two PA-specific epithelial (PASE) cell populations. Interestingly, PASE cells varied within tumours in their expression of gene signatures related to epithelial development (Cluster 0, C0), bone formation (Cluster 1, C1), immunity (Cluster 2, C2), metabolism (Cluster 3, C3), stemness (Cluster 4, C4), and the cell cycle (Cluster 5, C5). Of note, we observed that C4 cells had higher ‘differentiation’ potency and represented the starting state during PA development. Then, we revealed that CD36, a marker gene of C4 cells, was a functional cell surface marker for the enrichment of TICs in PA. In addition, activation of PI3K-AKT signalling was observed in CD36^+^ PA cells and inhibition of the PI3K-AKT pathway suppressed the initiation and growth of PA. Taken together, these results uncover the cellular heterogeneity and the origin of PA and highlight potential intracellar signals controlling the initiation and progression of PA, which serve as a resource for further developing novel therapeutic strategies to manage PA in the future.

## Results

### A single-cell atlas of the PA and parotid gland

**Fig. 1:**
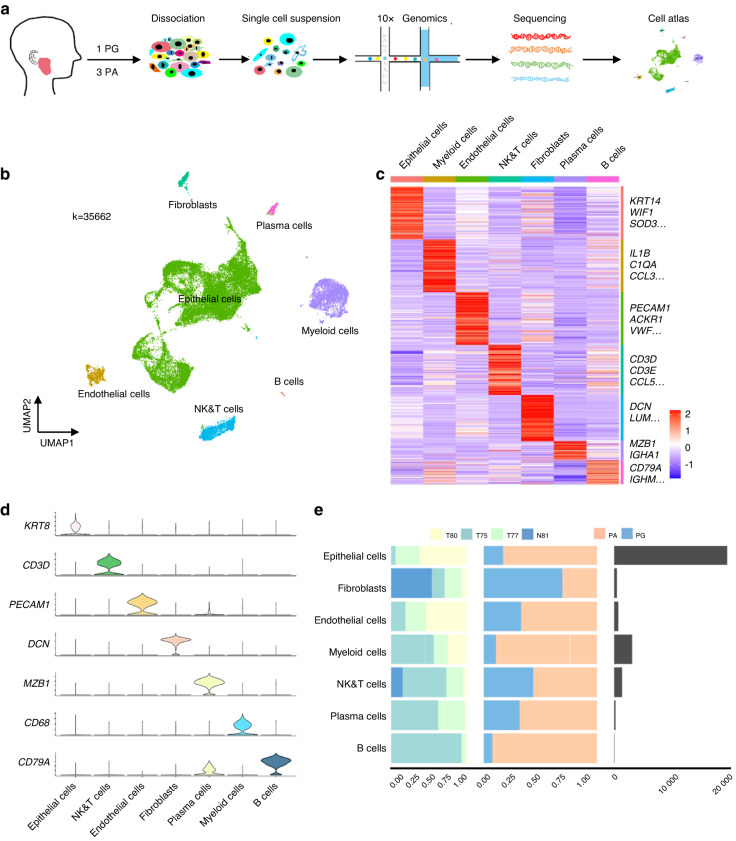
A single-cell atlas of PA and PG. **a** The workflow of single-cell RNA sequencing. **b** UMAP plot of 35 662 single cells from scRNA-seq labelled by cell type. **c** A heatmap of marker genes for each cell type. **d** StackedVlnPlot of classic genes of each cell type. **e** The cell numbers and proportions of PA cell types in different samples and tissue types

To explore the cellular landscape of PA, we generated single-cell RNA-seq profiles using 1 normal parotid gland (PG) and 3 PA samples (Fig. 1a). As shown in Fig. S1a, H&E staining showed that the pathological structure of PA varied in different samples, reflecting pleomorphic architecture features. After removing low-quality cells and performing gene expression normalization, a total of 35662 single cells were processed for further analysis and 8 cell clusters were observed according to graph-based clustering and dimensional reduction with UMAP (Fig. S1b, c). As shown in Fig. S1d, e, the UMAP displayed that the cell distribution was similar in different PA samples, but dramatically different in PA compared with the PG. This finding indicated that PA might have a unified cellular landscape despite the variable morphological architectures. Then, 7 major cell types were annotated based on the expression of canonical gene markers and assessment using the SingleR package (Fig. 1b–d, Fig. S2 and Supplementary Table 1), including epithelial cells expressing *KRT8, KRT14, KRT5, EPCAM*, *KRT18* and *AQP5*; endothelial cells expressing *PECAM1*, *ENG* and *CDH5*; fibroblasts expressing *COL3A1* and *DCN*; NK/T cells expressing *CD3D, CD3E, CD8A, NKG7* and *GNLY*; B cells expressing *CD79A* and *CD79B*; plasma cells expressing *MZB1*; and myeloid cells expressing *CD68, CD74, CLEC9A* and *CD163*. Of note, we observed that each of the 7 clusters contained cells from different samples despite differing cell type proportions, indicating that the major cell types are largely consistent across the PA and PG (Fig. 1e).

### Molecular characteristics of PA and PG epithelial cells

**Fig. 2:**
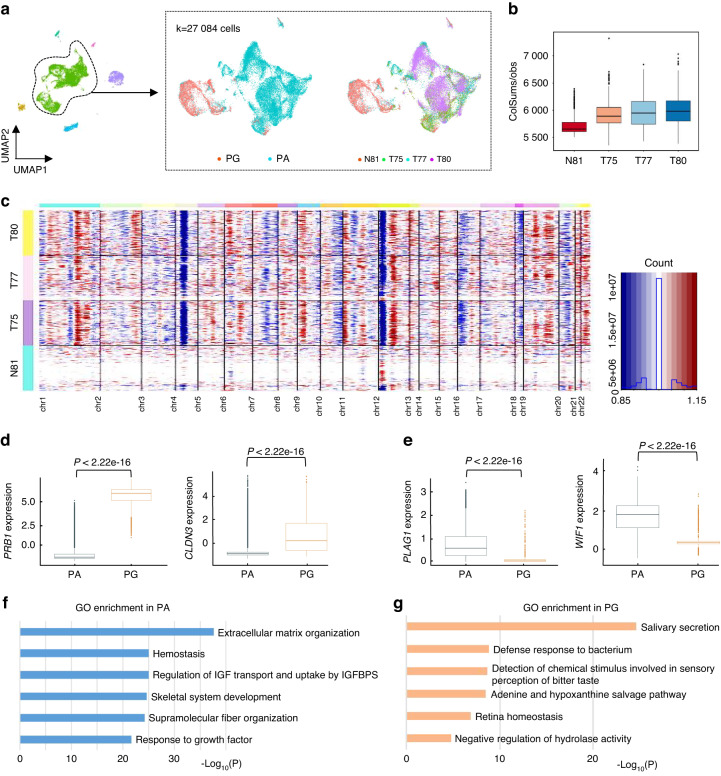
Molecular characteristics of PA and PG epithelial cells. **a** The UMAP distribution of 27 084 epithelial cells in different tissue types and samples. **b** Boxplot displaying the CNV score of epithelial cells in the four samples. **c** Chromosomal copy number variation values in epithelial cells of four samples, blue indicates deletion and red indicates amplification. **d** Boxplots showing the high differential expression of the representative genes *PRB1* and *CLDN3* in the PG sample (*P* < 2.2e-16). **e** Boxplots showing the high differential expression of the representative genes *PLAG1* and *WIF1* in the PA samples (*P* < 2.2e-16). **f** GO analysis of genes in PA epithelial cells (*P* < 0.05, log2FC > 1). **g** GO analysis of genes in PG epithelial cells (*P* < 0.05, log2FC > 1)

As shown in Fig. 2a, the UMAP showed a substantial difference in the distribution of epithelial cells from PG and PA. To characterize the PG and PA epithelial cells, we inferred copy-number variations (CNVs) in epithelial cells of each sample based on smoothed expression profiles across chromosomal intervals.^11^ We found that PA epithelial cells exhibited remarkably higher CNV levels than PG epithelial cells (Fig. 2b). The inferred CNV data analysis revealed significant copy number amplification on chromosomes 1, 9, 19, 22, but significant deletions along chromosomes 4, 16, 18 and 20 were observed (Fig. 2c). Of note, dramatic alterations were observed on chromosome 12. Similar genomic alterations were also observed in previous studies^12–14^ and several other adenogenic tumours, such as breast cancer and pancreatic ductal adenocarcinoma,^15,16^ indicating that adenogenic tumours might share some common genomic alterations. Subsequently, a panel of marker genes were identified to be upregulated in PA and PG epithelia cells. As expected, high expression of marker genes related to the secretion of salivary gland and saliva enzymes was observed in PG epithelial cells, such as *PRB1, CLDN3, PRB3, SMR3B, HTN1* and *PIGR*,^17,18^ while several oncogenes that have been confirmed to promote PA development were increased in PA cells, including *PLAG1, WIF1, S100B, CDK4, LIFR and NFIB* (Fig. 2d, e and Fig. S3a–c).^14,19–23^ Gene ontology (GO) enrichment analysis was performed based on the differentially expressed genes (DEGs) and revealed that matrix formation-related pathways were involved in the tumour epithelium, such as extracellular matrix organization and skeletal system development, suggesting that abnormal matrix formation occurred in the PA epithelium (Fig. 2f). In contrast, several pathways related to the physiological function of salivary glands were mainly enriched in the PG epithelium, including salivary secretion and detection of chemical stimuli involved in sensory perception of bitter taste (Fig. 2g)

### PA-specific epithelial cell subpopulations

**Fig. 3:**
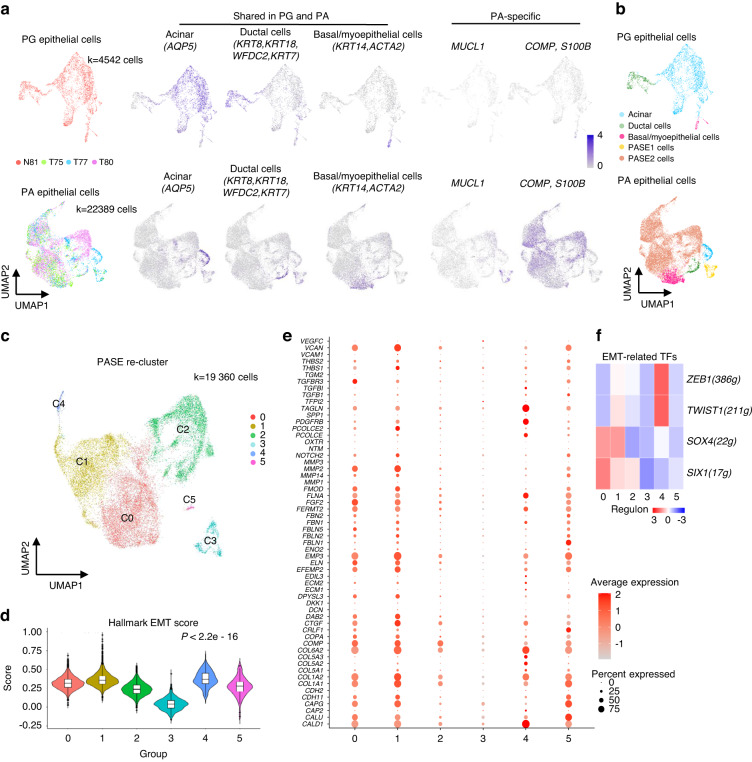
PA-specific epithelial cell subpopulations. **a** The UMAP distribution of epithelial cells labelled by samples (left) and the expression of acinar cells, ductal cells, basal/myoepithelial cell genes (middle) as well as the expression of tumour-specific genes (right) between PG and PA epithelial cells. **b** UMAP distribution showing the cell types shared with PG and PA samples and PA-specific cells. **c** The UMAP distribution of PA-specific epithelial cell subsets. **d** EMT score based on the HALLMARK_EPITHELIAL_MESENCHYMAL_TRANSITION gene set from GSEA datasets in different PA-specific epithelial cell subsets.^32^ *P* < 2.22e-16 by pairwise Wilcoxon rank-sum tests. **e** Dotplot showing EMT-related genes. **f** Heatmap showing the EMT-related transcription factors *ZEB1, TWIST1, SOX4* and *SIX1*

After reclustering PG epithelial cells, three major cell types were identified in the PG, including acinar cells expressing *AQP5*, ductal cells expressing *KRT18*, *WFDC2, KRT8*, and *KRT7* and basal cells/myoepithelial cells expressing *KRT14* and *ACTA2*, which were consistent with the known functional cells within the salivary gland.^24^ Similarly, reclustering of PA cells produced 5 major cell types and three of them were also observed in PG, including acinar cells (*AQP5*), ductal cells (*KRT18, KRT8, KRT7, WFDC2*) and basal/myoepithelial cells (*KRT14, ACTA2*), suggesting that PA tumour cells broadly recapitulated the PG cell subpopulations with shared several common marker genes across normal PG and PA (Fig. 3a). Interestingly, two distinguished epithelial cell clusters specific to PA were discovered, which were named the PA-specific epithelial cell (PASE) subpopulations and exhibited high expression o*f MUCL1* (PASE1) and *COMP* (PASE2) (Fig. 3b). Strikingly, PASE subpopulations accounted for 86.5% of tumour cells in PA.

To further investigate the heterogeneity and molecular characteristics of PASE cells, we performed analysis of 19360 PASE cells at higher resolution, which yielded 6 prominent cell subpopulations with different transcriptional profiles (C0-C5), reflecting the heterogeneity of PASE cells (Fig. 3c). It has been suggested that epithelial-mesenchymal transition (EMT) represents the basic principle of tissue heterogeneity in PA.^7–9^ Importantly, EMT has been suggested to be responsible for generating tumor-initiating cells in many tumors.^25–27^ Then, we first detected the EMT state in these six PASE cell populations and found that C0, C1, C2, C4 and C5 cells all exhibited high EMT scores (*n* = 200 genes, *P* < 2.2 × 10^−16^) (Fig. 3d). Notably, C4 cells had the highest EMT score but C3 cells had the lowest EMT score. These results indicated C4 cells were the undifferentiated cells, C3 cells were highly differentiated, and C0, C1, C2 and C5 cells showed the transition or intermediate states. Furthermore, all PASE subpopulations except C3 cells expressed high levels of EMT-related genes, such as *COL1A1, COL1A2, COMP, MMP2* and *EMP3* (Fig. 3e). Interestingly, several master transcription factors of EMT were also increased in C0, C1, C2 and C4 cells, including *SOX4, SIX1, ZEB1 and TWIST1* (Fig. 3f). These results suggest that EMT occurs widely and plays a critical role in PA development, which may contribute to the generation of TICs and the formation of stromal-like components, such as myxoid-like and chondroid-like components.

**Fig. 4:**
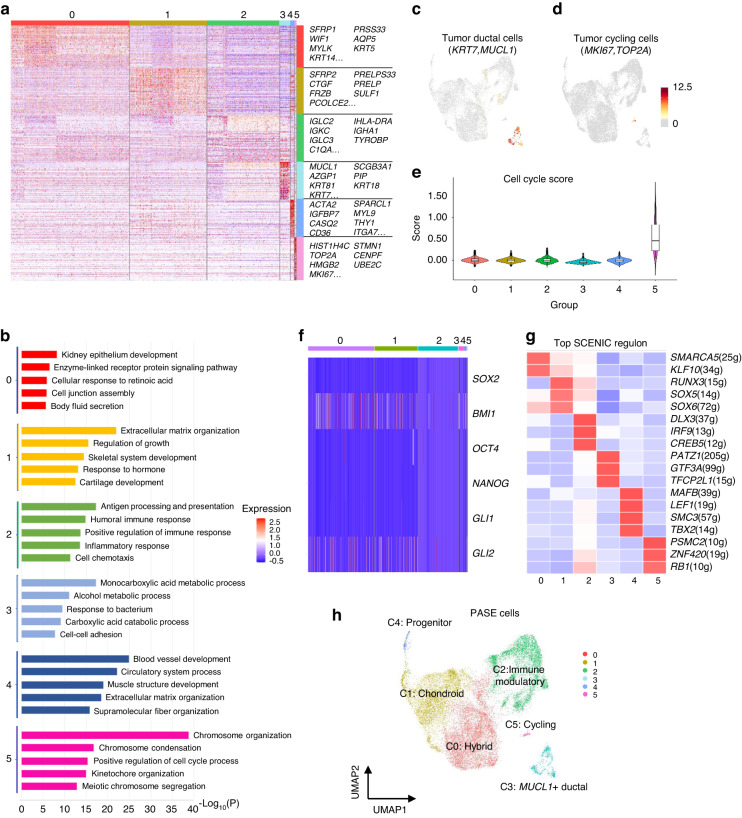
Transcriptional landscape heterogeneity of PA-specific epithelial cell subsets. **a** Heatmap of marker genes for PA-specific epithelial cell subsets. **b** GO enrichment analysis of PA specific epithelial cell subsets. **c** UMAP feature plots of expressed genes in tumour-specific ductal cells. **d** UMAP feature plots of expressed genes in cell cycle cells. **e** Cell cycle scores of PA specific epithelial cell subsets. **f** Heatmap showing stemness-related genes *SOX2, BMI1, NANOG, OCT4, GLI1* and *GLI2*. **g** Heatmap showing the top regulon of each cell cluster inferred by SCENIC. **h** The UMAP distribution of PA-specific epithelial cell subsets

Then, the transcription states of PASE subpopulations were annotated according to well-known marker genes and functional enrichment analysis. As shown in Fig. 4a, b and Supplementary Table 2, we observed that C0 cells expressed acinar cell markers (AQP5, *AQP1*) and basal cell markers (*KRT5*, *KRT14*)^28,29^ with properties of epithelium development and fluid secretion, indicating that C0 cells demonstrated a hybrid state. C1 cells showed high expression of *SFRP2* and *CTGF*, which have been reported to enhance osteogenic differentiation.^30,31^ Functional enrichment showed that C1 cells were correlated with extracellular matrix organization, bone development and cartilage development, implying that C1 cells were chondroid-like components. C2 cells expressed *IGLC2* and exhibited immune and inflammation signatures, implying that these cells might trigger immune reactions and inflammatory responses. Based on the features mentioned above, we defined C0, C1 and C2 cells as mesenchymal-like cells due to high EMT scores and the characteristics of stromal cells. In addition, C3 cells expressed *MUCL1* and the canonical ductal cell markers *KRT7, KRT18* and *KRT19* (Fig. 4c). Functional enrichment showed that C3 cells were characterized by metabolic reprogramming. C5 cells expressed high levels of proliferation-related genes (such as *MKI67* and *TOP2A)* and had the highest cell cycle score, which were also correlated with the cell cycle gene signatures (Fig. 4d, e). Notably, we observed that C4 cells exhibited high expression levels of myoepithelial cell markers (*ACTA2)*,^24^ myogenesis-related genes (*CASQ2*, *IGFBP7, MYL9*),^32–34^ angiogenesis-related genes (*SPARCL1*, *ITGA7, CALD1*)^35–37^ and stemness-related genes (*CD36, THY1, ITGA7)*.^38–43^ Functional enrichment showed that C4 cells were correlated with blood vessel development and muscle structure development, implying that C4 cells might have multidifferentiation potentials. However, C4 cells lacked expression of the classic stemness-related transcription factors, including *SOX2, BMI1, GLI1, NANOG*, and *OCT4* (Fig. 4f). Therefore, we performed single-cell regulatory network inference and clustering (SCENIC), which nominated *MAFB, LEF1* and *TBX2* as master TFs potentially controlling C4 cells (Fig. 4g), which have been confirmed to be associated with stemness and EMT.^44–46^ These findings indicated that the C4 subpopulation was a unique myoepithelial cell population with tumour-initiating potential in PA. Collectively, we then defined C0-C5 cells as hybrid, chondroid, immune modulatory, *MUCL1*^+^ ductal, progenitor and cycling PASE cells, respectively (Fig. 4h).

### Tumour-initiating cells in PA

**Fig. 5:**
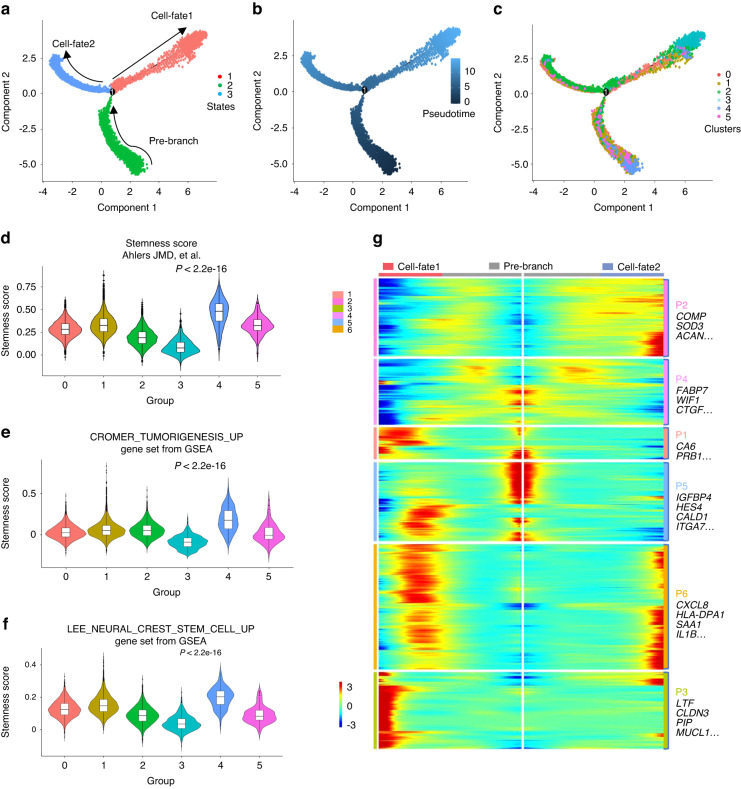
Tumour-initiating cells in PA. **a** The pseudotime trajectory is shown coloured by cell state designation. **b** Pseudotime is shown coloured in a gradient from dark to light blue and the start of pseudotime is indicated. **c** The pseudotime trajectory is shown coloured by cell subset designation. **d** The violin plot of stem cell score based on the published article^50^ (Supplementary Table 4) in different PA specific epithelial cell subsets (log2FC > 0.5). *P* < 2.22e-16 by pairwise Wilcoxon rank-sum tests. **e** Violin plot of stem cell score based on the CROMER_TUMORIGENESIS_UP gene set from GSEA datasets in different PA-specific epithelial cell subsets.^48^ *P* < 2.22e-16 by pairwise Wilcoxon rank-sum tests. **f** Violin plot of stem cell scores based on the LEE_NEURAL_CREST_STEM_CELL_UP gene set from GSEA datasets in different PA specific epithelial cell subsets.^49^ *P* < 2.22e-16 by pairwise Wilcoxon rank-sum tests. **g** Heatmap showing the ordering gene expression dynamics during the cellular-fate transition process and expression dynamics

Histologically, PA is characterized by a biphasic architecture with both “epithelial” and “stromal” regions.^47^ Currently, several studies have indicated that the epithelial and stromal elements are monoclonal and derived from the same precursor cells.^5,6^ However, it is still not clear which cell is responsible for tumour initiation and produces the epithelial and stromal components in PA. We showed that C4 PASE cells might have tumour-initiating potential in PA, as mentioned above. To further investigate the origins and developmental trajectory of PA cells, we performed trajectory analysis using the top 100 DEGs of PASE cells. This unsupervised approach identified a continuum of cell states and showed two distinct trajectories beginning at state 2 (pre-branch) and gradually branching to state 1 and state 3 corresponding two distinct cell fates (cell-fate 1 and cell-fate 2), revealing a common origin with divergent fates (Fig. 5a, b). In the pseudotime trajectory, we investigated the distribution pattern of the six PASE subtypes. As shown in Fig. 5c and Fig. S4a, C4 cells were predominantly observed in the end of state 2 with the lowest pseudo-time position and C3 cells were mostly found in the end of state 1 with the highest pseudo-time position, supporting our previous notion that C4 cells were the progenitor cells of PA and C3 cells were the highly differentiated ductal cells. C0, C1, C2 and C5 cells were scattered along the trajectories, reflecting their transition or intermediate states. Notably, the highly expressed gene markers of C4 (*ACTA2, IGFBP7, THY1*) were located at the low pseudotime position, further indicating that C4 cells were the progenitor cells in PA. The markers that represented the differentiated epithelial cells (*KRT19, EPCAM, KRT18*) and mesenchymal-like cells (*CNMD, COMP, SOX9*) were also located at the appropriate pseudotime position (Fig. S4b–d). Moreover, C4 cells exhibited high expression of the stemness-related gene signature and tumorigenesis-related gene signature by referring to gene sets that have been reported and included in GSEA^48–50^ (Fig. 5d–f). Taken together, these results suggested that C4 cells were the tumor-initiating cells in PA.

To study potential changes in global expression dynamics along the trajectory, we ordered the genes expression by pseudotime and conducted the enrichment analysis to investigate the precise impact of the alterations in cell fate. We found that there were 6 gene expression patterns (P1-P6) accounting for the distinctions (Fig. 5g, Supplementary Table 3). Cells undergoing cell-fate1 expressed highly homeostatic associated molecules enriched for the salivary gland function, including salivary secretion and monocarboxylic acid metabolic process (P1, P3). Cells undergoing cell-fate2 expressed high level of genes enriched for skeletal system development, extracellular matrix organization, inflammatory response and cytokine signaling in immune system (P2, P6), which were consistent with the signatures of mesenchymal-like cells mentioned above (Fig. 5g, Fig. S5). These functional enrichments reflected the cell components of PA in the two developmental routes, supporting the notion that TICs of PA could develop into the highly differentiated epithelial cells to form the duct structures and the mesenchymal-like cells with transitional states to form the stroma, including myxoid, chondroid or myxochondroid.

### CD36^+^ was a functional surface marker of tumour-initiating cells in PA

**Fig. 6:**
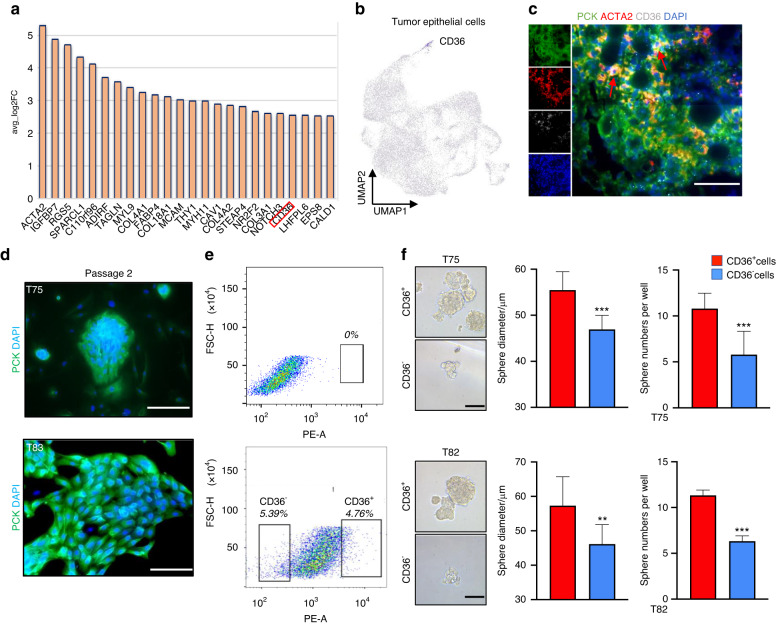
CD36^+^ is a functional surface marker of tumour-initiating cells in PA. **a** High expression genes of the C4 cluster (*P* < 0.05, avg_ log2FC > 2.5). **b** The UMAP distribution of *CD36* gene expression in PA epithelial cell subsets. **c** Representative images of multicolor immunostaining showing the expression of PCK, ACTA2, CD36 in PA sections; scale bar = 50 μm. **d** Immunofluorescence of PCK in PA epithelial cells after two passages culture; scale bar = 50 μm. **e** Flow cytometry analysis of CD36^+^ PA cells and CD36^−^ PA cells. **f** Spheroids cultured from CD36^+^ PA cells and CD36^−^ PA cells, scale bar = 50 μm (left). Quantification results are shown. **P < 0.01 and ***P < 0.001 by Student’s *t* test (right)

To further identify and isolate TICs of PA, C4 cell-specific markers were screened. Notably, we observed that CD36, a cell surface marker, was specifically expressed in PA epithelial cells (Fig. 6a, b) and involved in the regulation of stemness,^39,40^ indicating that CD36 might be a functional cell surface marker for the enrichment of TICs in PA. As shown in Fig. 6c, we further confirmed that CD36 was mainly expressed in myoepithelial tumour cells in PA tissue by using Pan-Keratin (PCK), ACTA2 and CD36 multiple immunostainings. Then, primary PA epithelial cells from different patients were freshly isolated and cultured, which were further confirmed using PCK staining (Fig. 6d). Next, CD36^+^ and CD36^−^ PA epithelial cells were sorted using fluorescence-activated cell sorting (FACS) (Fig. 6e). To evaluate the tumorigenic potential of sorted cells, a tumour sphere formation assay was performed using the collected CD36^+^ and CD36^−^ PA cells. As shown in Fig. 6f, CD36^+^ PA epithelial cells were able to form more and larger spheres than those generated from CD36^−^ PA epithelial cells. More importantly, we performed RNA-seq by using CD36^+^ and CD36^−^ cells isolated from primary PA cells. GSEA results showed that the genes upregulated in CD36^+^ cells were enriched for the regulation of stemness, supporting that CD36 was a cell surface marker for the enrichment of TICs in PA (Fig. S6a, b).

### Targeting the PI3K-AKT pathway eliminated tumour-initiating cells and suppressed tumour growth in PA

**Fig. 7:**
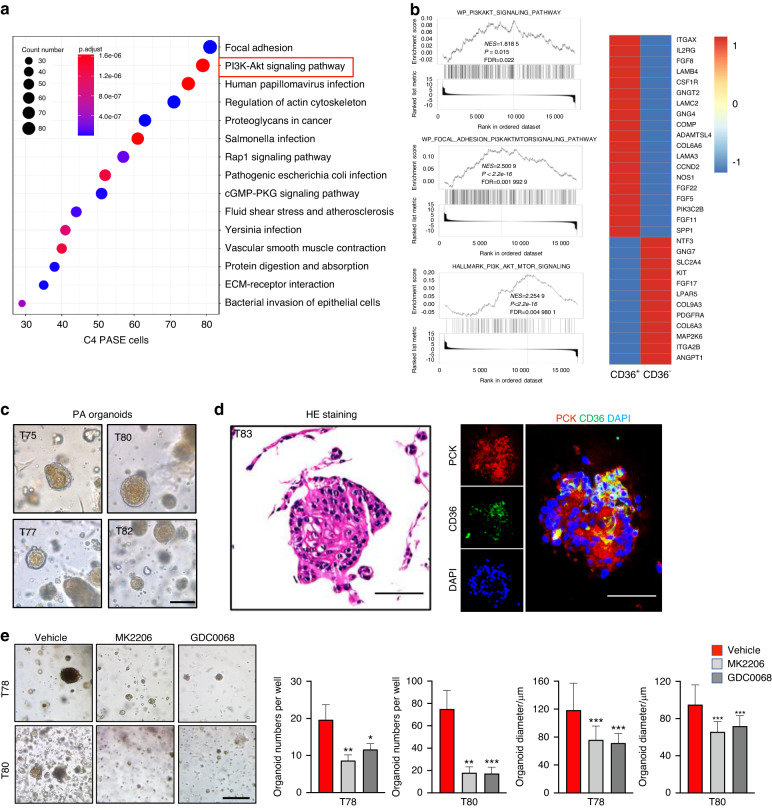
Targeting the PI3K-AKT pathway eliminated tumour-initiating cells and suppressed tumour growth in PA. **a** KEGG enrichment scatter plot of C4. The y-axis represents the name of the pathway, and the x-axis represents the rich factor, and the degree of KEGG pathway enrichment. The dot size represents the number of genes and the colour indicates the *q*-value. **b** GSEA showing that the PI3K-AKT pathway signatures were significantly enriched in CD36^+^ cells compared with CD36^−^ cells. The top DEGs (log2FC > 1) of these GSEA terms are presented in the heatmap. **c** Representative images showing patient organoids derived from different patient pleomorphic adenomas; scale bar = 100 μm. **d** Haematoxylin and eosin (H&E) staining of organoid sections; scale bar = 100 μm (left). Representative immunostaining images showing the expression of PCK and CD36 in PA organoids; scale bar = 100 μm (right). **e** The suppressive effect of AKT inhibitors (MK2206, 10 μmol·L^−1^ and GDC0068, 2.5 μmol·L^−1^) in the organoid assay, scale bar = 100 μm (left). Quantification results are shown. *P < 0.05, **P < 0.01 and ***P < 0.001 by one-way ANOVA (right)

Next, to clarify the key signalling pathway controlling TICs in PA, we performed Kyoto Encyclopedia of Genes and Genomes (KEGG) analysis based on our scRNA-seq data, which indicated that PI3K-AKT signalling pathway was activated in C4 cells (Fig. 7a). Similar results were also observed in our RNA-seq data generated from CD36^+^ and CD36^−^ PA cells (Fig. 7b). Then, two small molecular inhibitors MK2206 and GDC0068 were used to validate the functional role of the PI3K-AKT pathway in PA tumorigenesis. As shown in Fig. S7a, inactivation of the PI3K-AKT pathway with MK2206 and GDC0068 dramatically inhibited sphere formation generated from CD36^+^ PA cells. These findings suggested that targeting the PI3K-AKT pathway may be an effective therapeutic strategy for the treatment of PA. To further evaluate the therapeutic value of targeting the PI3K-AKT pathway in PA, PA patient-derived organoids (PPDOs) were cultured and confirmed by H&E staining and double staining of PCK and CD36 (Fig. 7c, d). As expected, we observed that pharmacological inhibition of the PI3K-AKT pathway significantly impaired the formation and growth of PPDOs (Fig. 7e).

## Discussion

The complex cytomorphological and architectural features of PA have been well recognized in past decades. However, the landscapes of cell constituents and their genetic heterogeneities are still mysterious and have never been documented. In the present study, we employed scRNA-seq technology to generate a comprehensive gene expression atlas of PA and identify seven major cell types with UMAP clustering that contribute to PA lesions. Notably, we showed that PA epithelial cells were dramatically different from their normal counterparts. High copy number variations were observed in PA epithelial cells compared to normal salivary epithelial cells despite the benign nature of PA. Obvious CNVs were detected on chromosomes 1, 4, 9, 12, 19 and 22, implying that the occurrence of PA results from multiple genomic alterations. It has been confirmed that PA is characterized by recurrent chromosome rearrangements, particularly translocations with breakpoints at 8q12 and 12q13-15, which activate the overexpression of *PLAG1* and *HMGA2*.^14,51^ In our study, the predominant copy number alterations were observed in chromosome 12, in which some key genes driving tumorigenesis of PA were located, including *HMGA2*, *WIF1*, *MGP* and *MUCL1*. These findings suggest that the alteration of chromosome 12 may be the genomic hallmark of PA.

Then, we characterized the features of gene expression profiles in PA epithelial cells and revealed intratumor heterogeneity and diversity. Consistent with a previous study in cutaneous squamous cell carcinoma,^52^ we observed that several PA tumour subpopulations (acinar, ductal and basal/myoepithelial cells) were also similar to their normal counterparts, which shared several marker genes across normal PG and PA, suggesting that PA might recapitulate stages of PG development. Strikingly, two PA subpopulations unique to tumour were identified, which accounted for most of the tumour cells in PA. Then, we identified six subgroups (C0–C5) with different transcriptomic characteristics in tumour specific epithelial cells. C0, C1, C2, C4 and C5 cells were all characterized with properties of EMT but exhibited different differentiation degrees and proliferative activities, indicating that EMT was widely activated during the tumorigenesis of PA. C0 cells were associated with epithelial development, C1 cells were correlated with chondrogenesis and osteogenesis, and C2 cells displayed dual characteristics of epithelial cells and immune cells, revealing a high degree of tumor heterogeneity. In addition, C3 was a ductal cell population with a low EMT score and high expression of *MUCL1* gene, which demonstrated obvious epithelial features and might form the luminal structures of PA. Increasing studies have confirmed that *MUCL1* functions as a unique oncogene to promote proliferation and metastasis in several adenogenous malignancies, including breast cancer,^53^ gastric adenocarcinoma,^54^ and colorectal adenocarcinoma,^55^ indicating that the *MUCL1* gene may be a potential marker to evaluate the aggressiveness of PA. However, further studies are required to examine the expression of *MUCL1* and *MUCL1*^+^ ductal cell subpopulations in PA.

It has been suggested that TICs or tumour stem cells are responsible for tumour recurrence, metastatic spread and resistance to treatment.^56–58^ Notably, we revealed that the C4 cluster housed the TICs based on single-cell algorithms rather than relying on predefined surface markers, which often target unrelated cells.^59,60^ Then, we confirmed that the cells within C4 showing high expression levels of *CD36* were the tumour stem cells resulting in the formation of PA. Similar results were also observed in glioblastoma,^39^ oral carcinomas^40^ and leukaemia.^61^ Next, we further revealed that the PI3K-AKT pathway was activated in C4 cells and CD36^+^ tumour cells, and inhibition of PI3K-AKT signalling suppressed PA tumorosphere formation and organoid growth. Currently, accumulating evidence has confirmed that the PI3K-AKT pathway plays an important role in cell mitosis, proliferation and apoptosis, especially in maintaining the pluripotency of mesenchymal stem cells and embryonic stem cells.^62,63^ Importantly, previous studies have shown that CD36 promotes tumour progression by activating PI3K/AKT in several cancer types.^64–67^ These findings support the notion that TICs of PA are controlled by PI3K-AKT signalling.

Taken together, our findings provide the first resource for deciphering comprehensive gene expression landscapes of PA and reveal the intratumoral heterogeneity and origin of PA at a single-cell resolution despite the small number of patients enrolled. Furthermore, we identified a unique myoepithelial cell population with high expression of CD36 and activation of PI3K-AKT signalling that contributes to tumorigenesis of PA, which provides a novel therapeutic target and lays a new foundation for the development of precision therapies in PA.

## Materials and methods

### Salivary tumour specimens

In compliance with all relevant ethical regulations, we collected fresh pleomorphic adenoma and adjacent nontumour tissue from patients at the Hospital of Stomatology, Sun Yat-sen University. Briefly, freshly harvested tissues were mechanically and enzymatically digested into single cell suspensions with gentleMACS (Miltenyi) according to the instructions of a tumour dissociation kit (Cat #130-095-929, Miltenyi Biotec, Bergisch Gladbach, Germany). Then, the dissociated cells were filtered with a 70-μm SmartStrainer and centrifuged at 400 × *g* for 5 min to remove large pieces of debris. After the supernatant was removed, the pelleted cells were incubated with red blood cell lysis buffer (Thermo Fisher Scientific, Carlsbad, CA) for 1–2 min to lyse red blood cells. Finally, the cells were washed twice with 1×PBS (GIBCO), and the cell pellets were counted and tested for cell viability using trypan blue staining.

### Tissue processing

The remaining tissues were fixed in 4% paraformaldehyde (Biosharp, BL539A) for 48 h at 4 °C overnight. Dehydration and embedding in paraffin were performed using routine methods. Paraffin sections (5 μm) were cut and adhered to glass slides. Then, the paraffin sections were placed at the room temperature.

### Haematoxylin and eosin (H&E) staining

The paraffin sections were placed in a 70 °C oven for 15 min before deparaffinization in xylene and successively hydrated in 100%, 90%, 80% and 70% alcohol. H&E staining was performed according to a standard protocol (Solarbio, G1120-100), and stained slides were dehydrated in ethanol and sealed in neutral resin.

### Immunofluorescence staining

Formalin-fixed paraffin-embedded sections of PA specimens were collected at the Hospital of Stomatology, Sun Yat-sen University. To detect the protein expression levels of marker genes, the PA paraffin sections were stained four-color-multilabeled immunofluorescence staining kit (Absin, Cat#abs50012) according to the manuffacture’s protocols. Briefly, the sections were incubated in 3%H_2_O_2_ for 10 mins for the first time. Every time the sections were incubated with antibodies against CD36 (ab17044, Abcam), Pan-keratin (PCK) (26411-AP, Proteintech), ACTA2 (ab220179, Abcam). After each incubation of the primary antibody, heat-induced epitope recovery and 5% BSA blocking were performed. The HRP conjugate and three wavelengths (520,570 and 650 nm) were utilized to attach the different primary antibodies. Then, the slides were counterstained with DAPI for nuclear visualization, and subsequently coverslipped with fluorescence quenching mounting medium. Images were acquired using a fluorescence microscope. The organoid immunofluorescence staining was performed as described previously with antibodies against CD36 and PCK^68,69^. All primary antibodies were used at a dilution of 1:200.

### 10× library preparation and sequencing

According to the standard manufacturer’s protocol, single cells were resuspended in DMEM buffer at 1 000 cells per μL and loaded onto the Chromium chips. All the remaining procedures including capturing, barcoding and cDNA library preparation were carried out using the Chromium Single Cell 3’Library v3 chemistry (10x Genomics).

### Single cell RNA-seq data processing

Sequenced reads were aligned and quantified using the Cell Ranger 4.0.0 pipeline against the GRCh38 human reference genome. Next, by counting unique molecular identifiers (UMIs) and removing low quality barcodes, a gene barcode matrix containing the barcoded cells and gene expression counts was generated. The expression matrix passed the quality control based on three metrics step by step, including the total cell count, number of detected genes and the proportion of mitochondrial gene count per cell, by using the Seurat R package (v4.0)^70,71^ to generate Seurat Objects for downstream analyses. High quality cells (200 < nFeature_RNA < 9000, nCount_RNA > 1000 and percent. mt < 10) were included. Then, the following Seurat functions were performed on remaining 35662 cells using the scran and scater packages in R.^72^

### Integration of multiple scRNA-seq datasets

We ran the CCA^+^ anchors, an algorithm originating from Seurat v3.0 that can identify the shared cell state among different scRNA-seq datasets to remove the batch effects of 4 samples. The results from CCA integration and batch correction were used as input data for highly-variable gene identification and dimension reduction.

### Dimension reduction and unsupervised clustering

Single-cell data were processed for dimension reduction and unsupervised clustering by following the workflow in Seurat v3.0. In brief, 2 000 highly variable genes were summarized by principal component analysis (PCA) and a total of 20 principal components were selected for dimensional reduction using the default settings of the RunPCA function. Finally, the dimensionality of each dataset was further reduced using uniform manifold approximation and projection (UMAP) for visualization.

### Major cell types determination and marker genes identification

Doublets with mix features were deleted from further analysis using the DoubletFinder (v2.0.3) function. The remaining cells were used for the downstream analysis. We determined cell types by using Seurat package in combination with the SingleR (v1.4.1) package. The specific gene markers were identified using the FindAllMarkers function. Cell clusters were annotated as biological cell types according to canonical marker genes. To refine the classification of various cell types, we further compared our cell-type annotation to the reference by SingleR (v1.4.1).

### Subclustering of some major cell types

To identify subclusters with some major cell types, we reclustered cells belonging to epithelial cells, myeloid cells and NK/T cells separately using the workflow in Seurat v3.0. Applying the graph-based clustering approach, with a unique resolution and other default parameters, they were reclustered by its principal components. For visualization purposes, these informative principal components were converted into UMAP plots. To further investigate the characteristics of various clusters, cluster marker genes were detected using the FindMarkers function.

### CNV estimation based on scRNA-seq data

Initial CNVs for tumour epithelial cells of each sample were calculated by the inferCNV R package.^73^ For each sample, the CNV from the single-cell RNA-seq dataset was explored by the expression intensity of genes across positions of the genome compared with a set of reference cells. We set 1 000 cells from normal samples for reference, and 1 000 cells each from other tumour samples for observations and ran inferCNV with cut-off = 0.1. The expected output is a heatmap of observed expression relative to the reference, shedding light on regions of chromosomal gain and loss.

### EMT score, cell cycle score and stemness score

According to the reference gene sets, subsets were scored using the Seurat function AddMouduleScore. We calculated the mean abundance levels of the subset function-related mRNA genes against the abundance of the control gene sets as the score of each epithelial subset.

### Delineation of cell differentiation trajectories

To uncover the cell state transition, the Monocle package^74^ was applied to depict single cell trajectories. Briefly, the Seurat Object was converted into the Monocle cell dataset using the ‘importCDs’ function. The top 100 differentially expressed genes in the subclusters of epithelial cells were selected using the differential-GeneTest function in pseudotime order. We then applied ‘DDRtree’ to reduce dimensions and the visualization functions ‘plot_cell_trajectory’ to plot the minimum spanning tree on cells.

### Analysis of transcription factor expression

To assess the transcriptional activity of tumour-specific epithelial cells, single-cell regulatory network inference and clustering (SCENIC) analysis was used to map the gene regulatory network and identify stable cellular states by evaluating the activity of GRNs (Gene Regulatory Networks) in each cell. The analysis used the 20-thousand motifs database for RisTarget and GRNboost (corresponding to GENIE3 1.4.3, AUCell 1.4.1 and RisTarget 1.2.1; with hg19.motifDatabases.20k).^75^ The input matrix was normalized expression matrix, output from Seurat.

### Metascape analysis GO enrichment

We performed the analysis via website (https://metascape.org/gp). Gene sets were input into the Metascape database for GO enrichment analysis.

### Flow cytometry

Fresh pleomorphic adenoma samples obtained from patients were digested with collagenase IV (Thermo Fisher Scientific, 17104019) and a single-cell suspension was acquired. Then, we cultured the cells in 10 cm petri dishes with common medium. After two passages, tumour epithelial cells were purified. Next, we obtained a single-cell suspension. Cells were sorted by PE anti-human CD36 antibodies (Biolegend, Cat#336205) with FACS Aria I (BD Biosciences, Germany).

### Sphere culture

The sorted cells were seeded at 5 000 cells per μL in low-attachment 96-well plate which added into DMEM/F12 (Thermo Fisher Scientific, Cat#C11330500BT), supplemented with 20 ng·mL^−1^ human EGF (PeproTech, Cat#AF-100-15), 20 ng·μL^−1^ FGF2 (Sino Biological Inc, Cat#10014-HNAE) and 1×B27 supplement (Thermo Fisher Scientific, Cat#12587010). Cells were cultured for 7 days before harvesting. Images were acquired using an inverted microscope.

### Organoid culture

For organoid culture, the tumour suspensions were seeded at 1 × 10^4^ cells per well in 200 μL Matrigel (Corning, 354234) in 24-well plates. After polymerization, organoids were cultured in self-configured medium containing DMEM/F12 (Thermo Fisher Scientific, Cat#C11330500BT), 1×B27 supplement (Thermo Fisher Scientific, Cat#12587010), 1.25 mmol·L^−1^ N-acetyl-L-cysteine (Sigma, Cat#A7250), 10 mmol·L^−1^ nicotinamide (Sigma, Cat#N0636), 50 ng·mL^−1^ human EGF (PeproTech, Cat#AF-100-15), 500 nmol·L^−1^ A83-01 (PeproTech, Cat#9094360), 10 ng·mL^−1^ human FGF10 (PeproTech, Cat#100-26-5), 5 ng·mL^−1^ human FGF2 (Sino Biological Inc, Cat#10014-HNAE), 1 μmol·L^−1^ prostaglandin E2 (MCE, Cat#HY-101952), 0.3 μmol·L^−1^ CHIR 99021 (Sigma, Cat#SML1046), 1 μmol·L^−1^ forskolin (Abcam, Cat#ab120058), 50 ng·mL^−1^ R-spondin (R&D Systems Cat#3266-RS) and 25 ng·mL^−1^ Noggin (PeproTech, Cat#120-10 C). The medium was changed twice a week.

### Statistical analysis

Data are shown as the mean ± SD. Statistical analyses were conducted by using Prism 8.0 (Graphpad Software). Statistical comparisons were performed using Student’s test or one-way ANOVA. *P* < 0.05 was considered to be statistically significant.

### Supplementary information

Figure S1

Figure S2

Figure S3

Figure S4

Figure S5

Figure S6

Figure S7

Supplementary Table1

Supplementary Table2

Supplementary Table3

Supplementary Table4

Supplementary figures legend

## Data Availability

The raw sequencing data of human PA tissue and cells have been deposited in GSA-Human under accession number HRA003970, including scRNA-seq data and RNA-seq data. All the other data associated with this study are available in the article and its supplementary information files and from the corresponding author upon reasonable request.
