# Supplementary figures and images for: Single-cell transcriptomic analysis uncovers the origin and intratumoral heterogeneity of parotid pleomorphic adenoma
Source: Int J Oral Sci. 2023 Sep 7;15:38. doi: 10.1038/s41368-023-00243-2 (PMC10484943; doi:10.1038/s41368-023-00243-2)

Figure S1

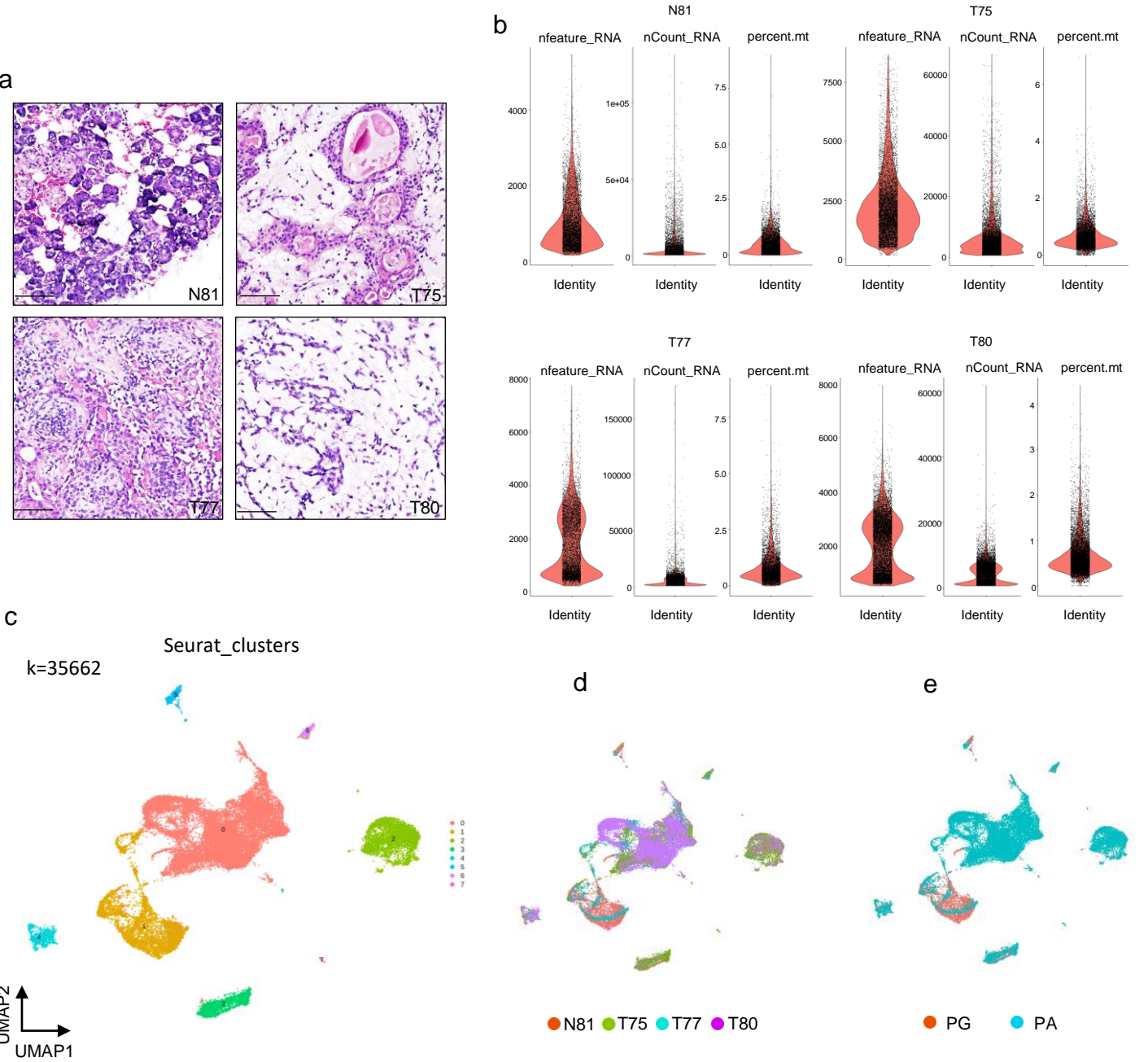

Supplement: Supplementary file 1 — Figure S1 [file 41368_2023_243_MOESM1_ESM.pdf]

Figure S2

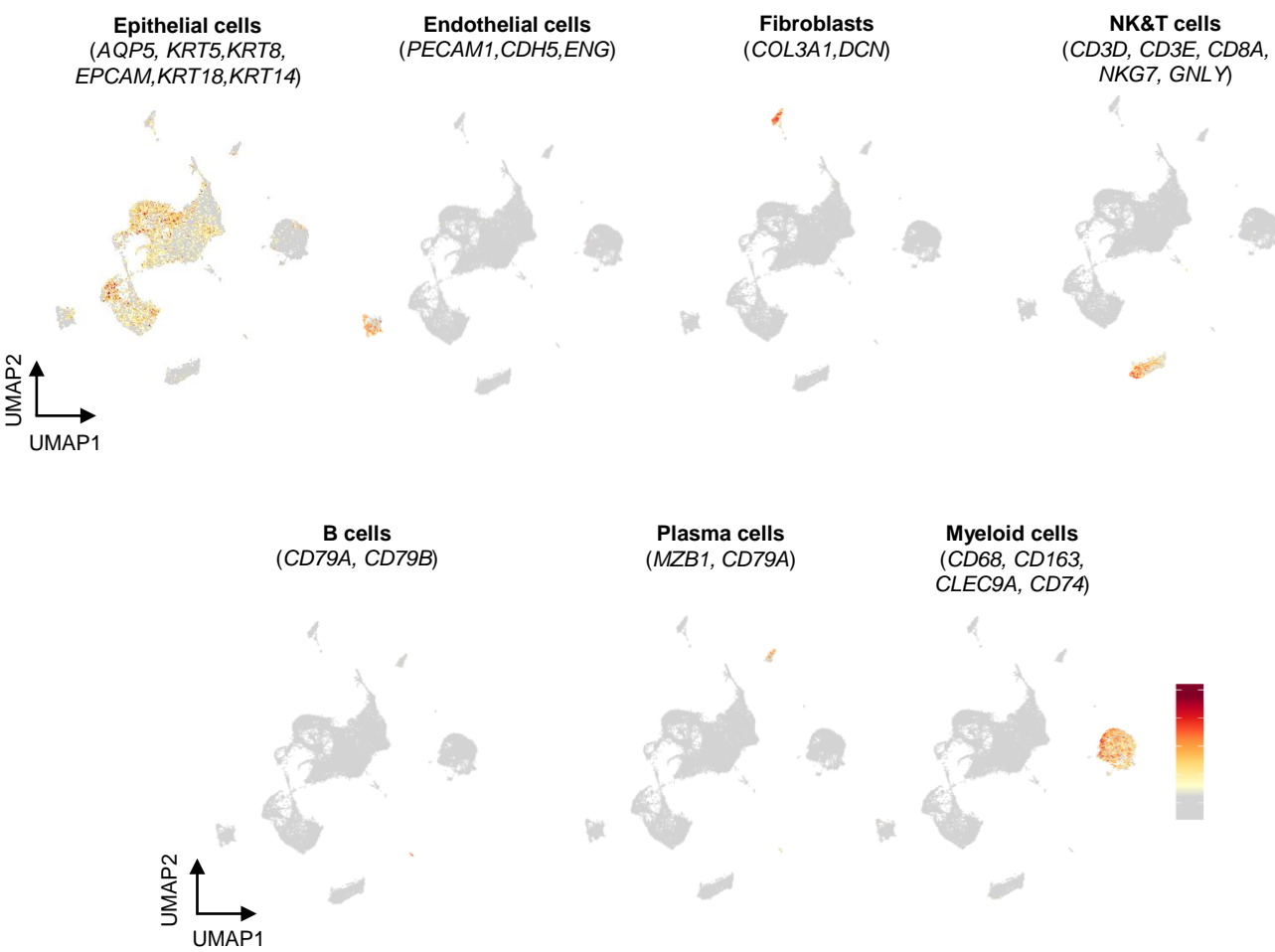

Supplement: Supplementary file 2 — Figure S2 [file 41368_2023_243_MOESM2_ESM.pdf]

Figure S3

a

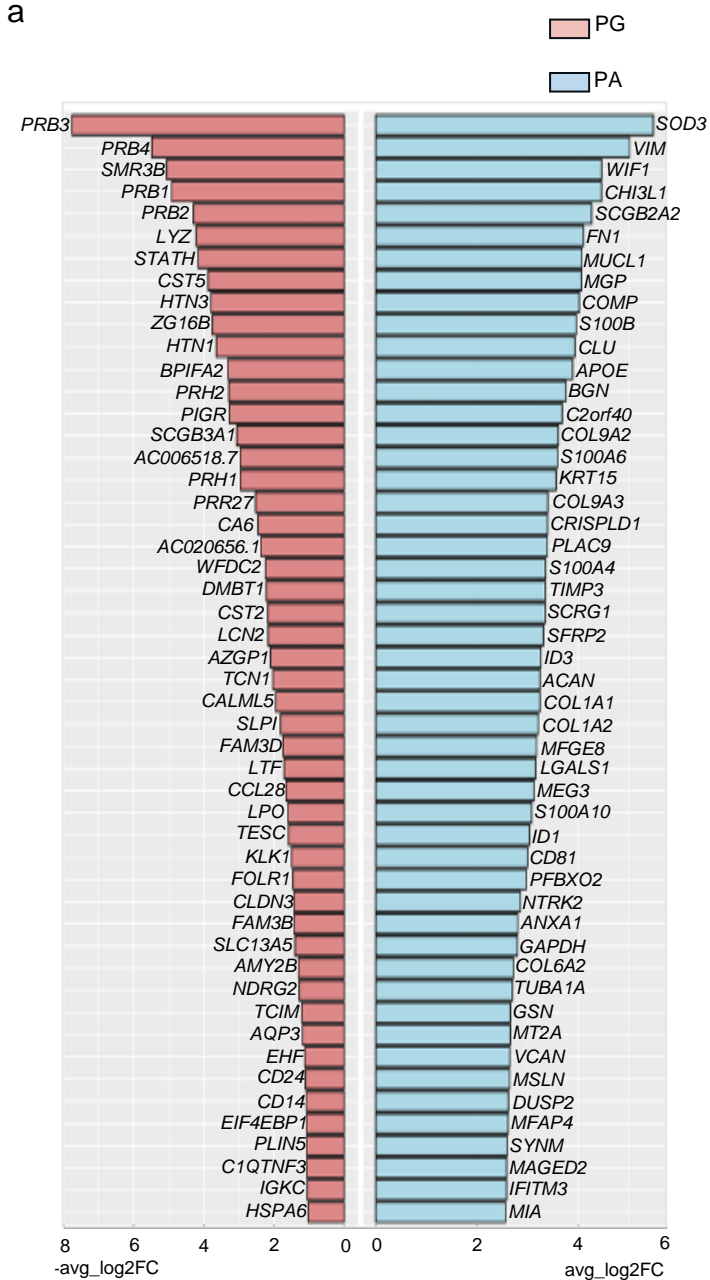

b

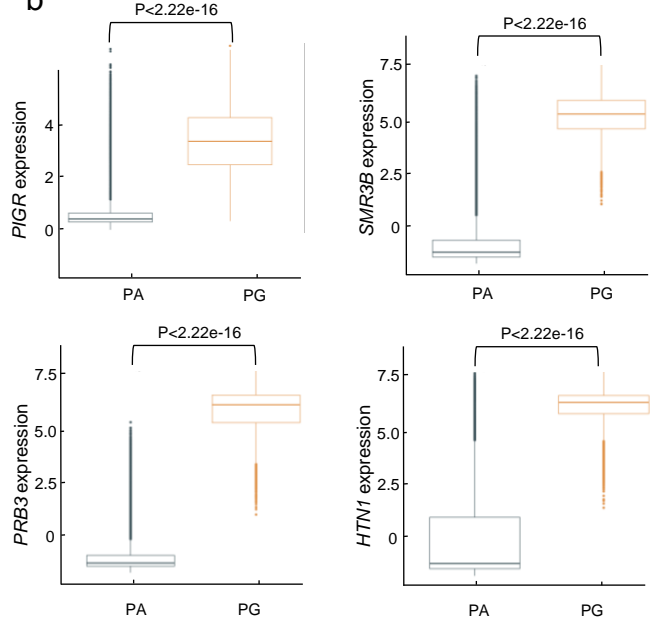

c

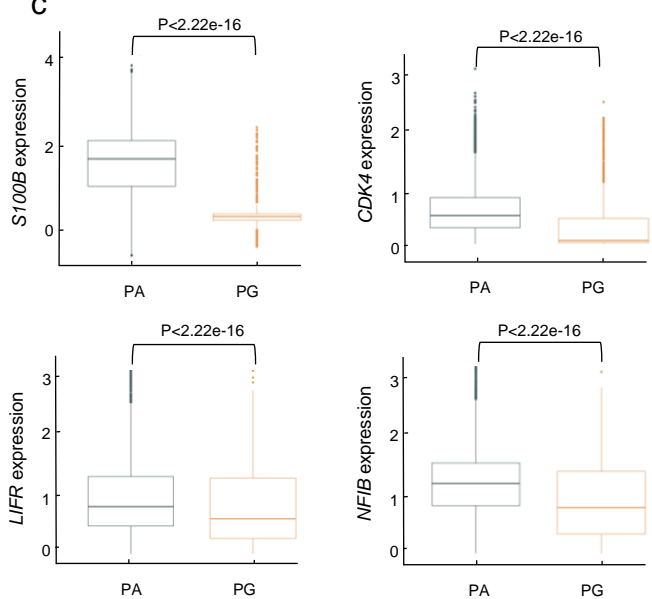

Supplement: Supplementary file 3 — Figure S3 [file 41368_2023_243_MOESM3_ESM.pdf]

Figure S5

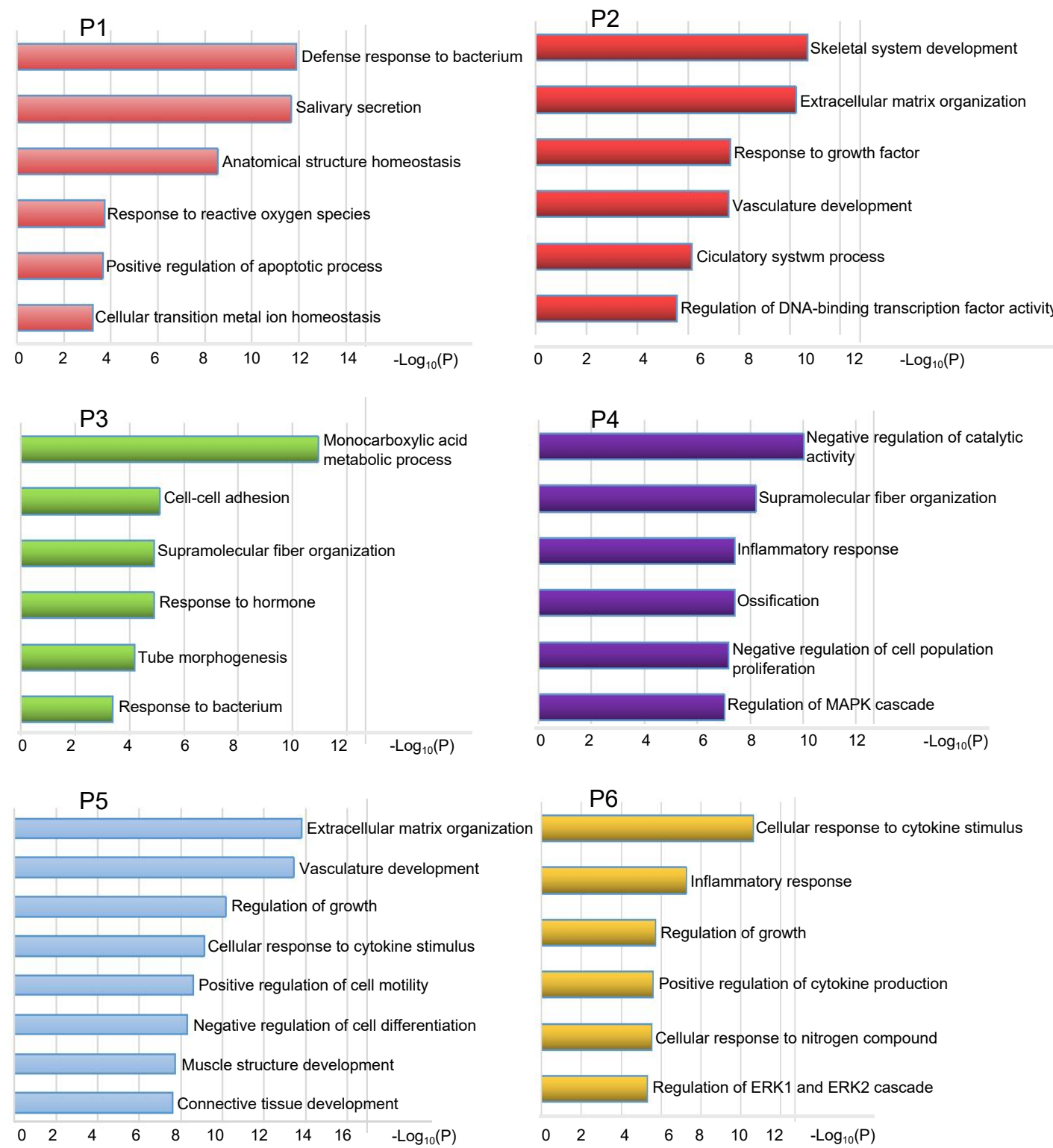

Supplement: Supplementary file 5 — Figure S5 [file 41368_2023_243_MOESM5_ESM.pdf]

Figure S6

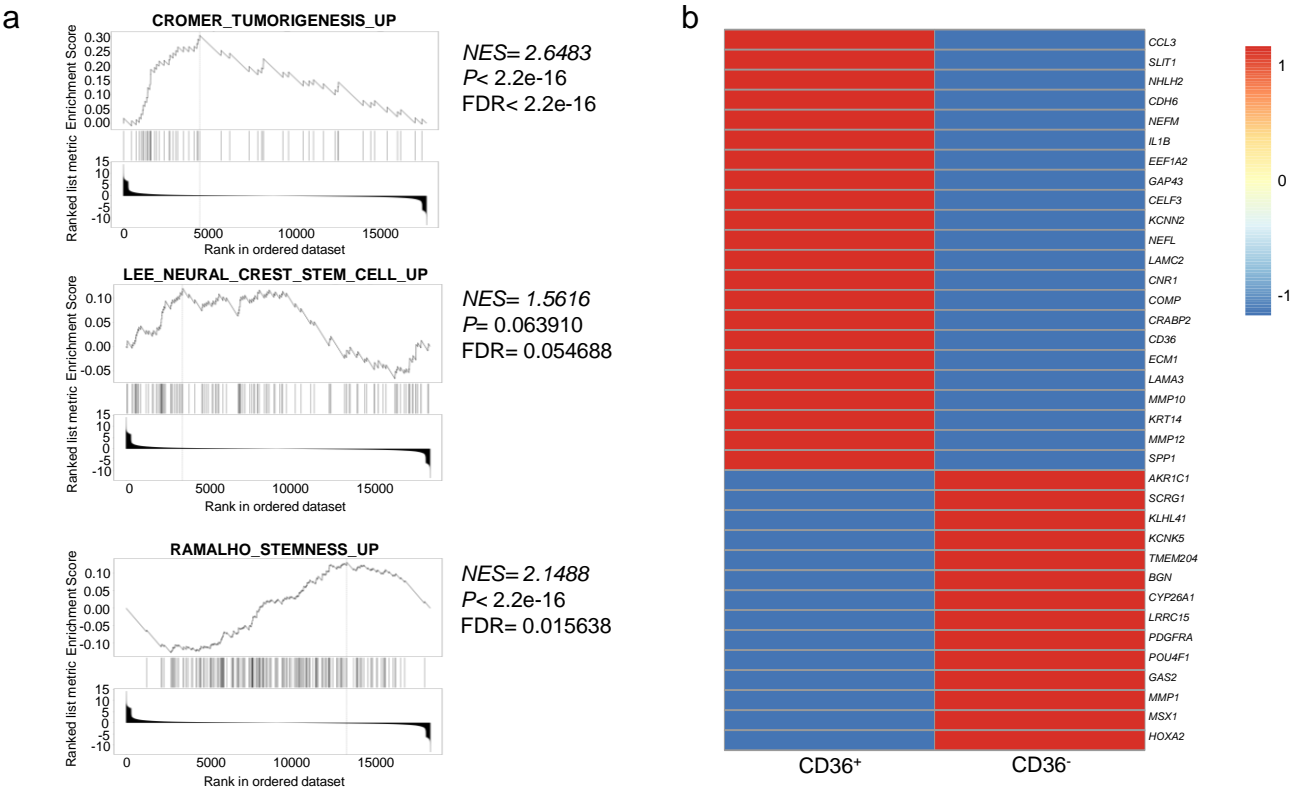

Supplement: Supplementary file 6 — Figure S6 [file 41368_2023_243_MOESM6_ESM.pdf]

Figure S7

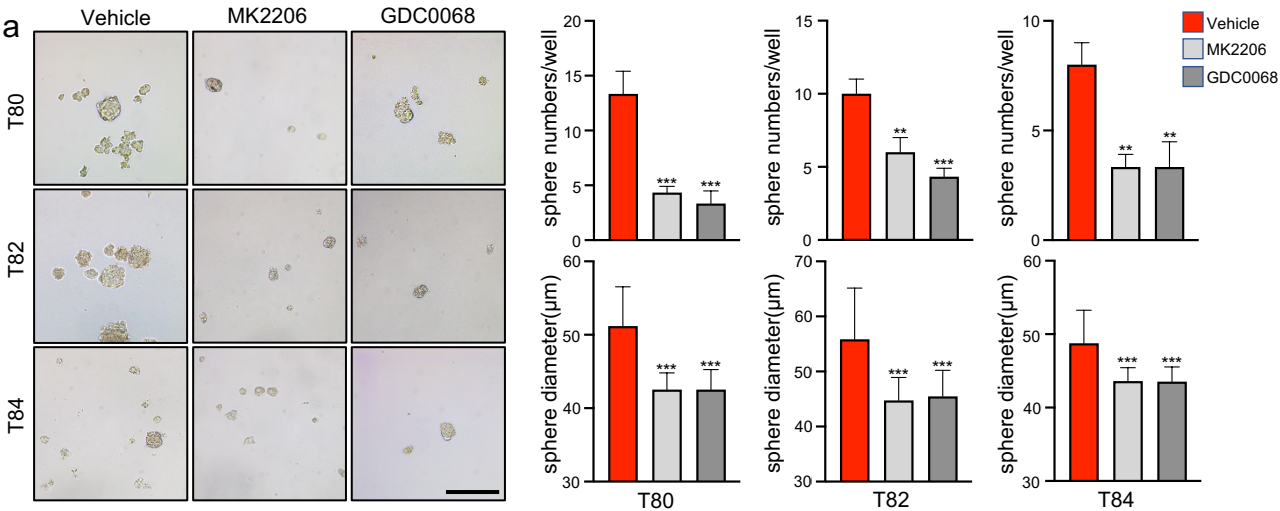

Supplement: Supplementary file 7 — Figure S7 [file 41368_2023_243_MOESM7_ESM.pdf]
