# Supplementary material for: Single-cell transcriptomic analysis uncovers the origin and intratumoral heterogeneity of parotid pleomorphic adenoma
Source: Int J Oral Sci. 2023 Sep 7;15:38. doi: 10.1038/s41368-023-00243-2 (PMC10484943; doi:10.1038/s41368-023-00243-2)
Supplement: Supplementary file 4 — Figure S4 [file 41368_2023_243_MOESM4_ESM.pdf]

Figure S4

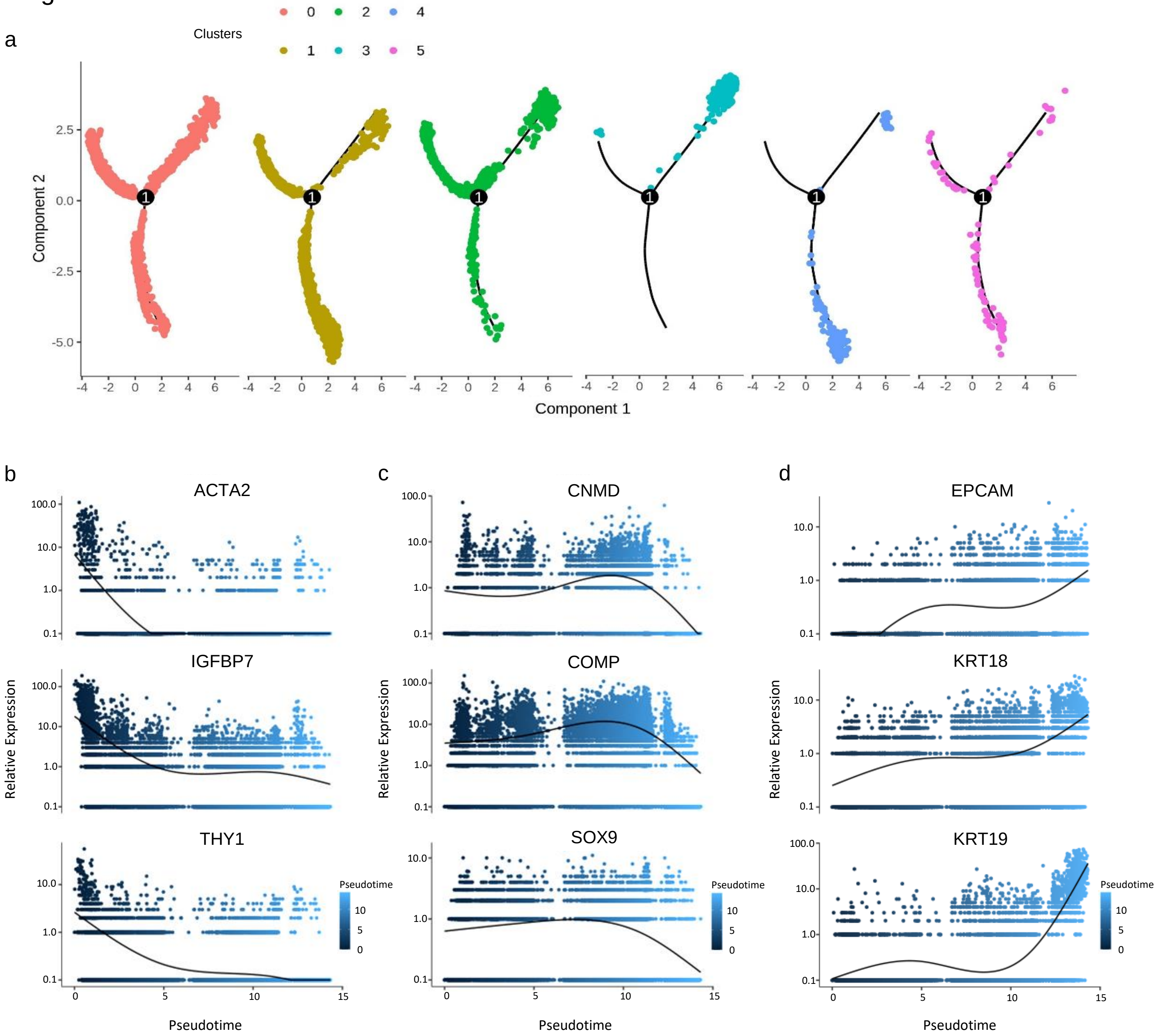

a
Clusters
0
1
2
3
4
5
Component 2
Component 1
2.5
0.0
-2.5
-5.0
1
b
ACTA2
IGFBP7
THY1
c
CNMD
COMP
SOX9
d
EPCAM
KRT18
KRT19
Relative Expression
Pseudotime
100.0
10.0
1.0
0.1
0
5
10
15
