# Supplementary material for: Single-cell transcriptomic analysis uncovers the origin and intratumoral heterogeneity of parotid pleomorphic adenoma
Source: Int J Oral Sci. 2023 Sep 7;15:38. doi: 10.1038/s41368-023-00243-2 (PMC10484943; doi:10.1038/s41368-023-00243-2)
Supplement: Supplementary file 12 — Supplementary figures legend [file 41368_2023_243_MOESM12_ESM.docx]

**Supplementary Figures**

**Fig. S1, related to Fig. 1, The quality control and cell type of all samples.**

1. H&E staining of PA and PG tissues; scale bar=100 μm.
2. Quality control of all samples.
3. The UMAP distribution of 35662 cells labelled by cell clusters.
4. The UMAP distribution of 35662 cells labelled by different samples.
5. The UMAP distribution of 35662 cells labelled by PG and PA.

**Fig. S2, related to Fig. 1, UMAP feature plots of marker genes in different cell types.**

**Fig. S3, related to Fig. 2, The differential gene expression in PG and PA samples.**

1. Top 50 differentially expressed genes between PG and PA epithelial cells.
2. Box plots showing the high differential expression of the representative genes *PIGR, PRB3, HTN1* and *SMR3B* in the PG sample (*P* < 0.05).
3. Box plots showing the high differential expression of the representative genes *CDK4, S100B, LIFR* and *NFIB* in PA samples (*P* < 0.05).

**Fig. S4, related to Fig. 5, Pseudotime expression levels of representative genes**.

1. Distributions of C0-C5 clusters in pseudotime trajectory.
2. The marker gene expression level of C4 (*ACTA2, IGFBP7, THY1*) along the pseudotime axis.
3. The marker gene expression level of mesenchymal-like cells (*CNMD, COMP, SOX9*) along the pseudotime axis.
4. The marker genes expression levels of epithelial cells (*EPCAM, KRT19, KRT18*) along the pseudotime axis.

**Fig. S5, related to Fig. 5, The significant gene ontology (GO) terms for each pattern (P1-P6).**

**Fig. S6, related to Fig. 6, The RNA-seq characteristics of CD36^+^ cells in PA**

1. GSEA showing that the stemness signature was enriched by the DEGs between CD36^+^ cells and CD36^-^ PA cells. GSEA enrichment results for CROMER_TUMORIGENESIS_UP, LEE_NEURAL_CREST_STEM_CELL_UP and RAMALHO_STEMNESS_UP.
2. Heatmap showing the gene expression profiles of CD36^+^ and CD36^-^ cells from RNA-seq. CD36 and the top DEGs (log2FC>1) related to stemness, myoepithelial, EMT and GSEA terms are presented.

**Fig. S7, related to Fig. 7, Targeting the PI3K-AKT pathway inhibited tumour growth in PA**

1. Sphere formation assays revealed the suppressive effect of the AKT inhibitors (MK2206, 10 μM and GDC0068, 2.5 μM), scale bar=100 μm (left). Quantification results are shown. ***P* < 0.01 and *** *P* <0.001 by one-way ANOVA (right).

**Supplementary Table 1. List of marker genes for each cell cluster in PA and PG.**

**Supplementary Table 2. List of marker genes for each cluster in PASE cells.**

**Supplementary Table 3. List of differentially expressed genes over the pseudotime from prebranch to cell fate1/2 transition.**

**Supplementary Table 4. The stemness-related gene set from reference 50.**
